# A geraniol synthase regulates plant defense via alternative splicing in tea plants

**DOI:** 10.1093/hr/uhad184

**Published:** 2023-09-12

**Authors:** Hao Jiang, Mengting Zhang, Feng Yu, Xuehui Li, Jieyang Jin, Youjia Zhou, Qiang Wang, Tingting Jing, Xiaochun Wan, Wilfried Schwab, Chuankui Song

**Affiliations:** State Key Laboratory of Tea Plant Biolog and Utilization, Anhui Agricultural University, 130 West Changjiang Road, Hefei 230036, China; State Key Laboratory of Tea Plant Biolog and Utilization, Anhui Agricultural University, 130 West Changjiang Road, Hefei 230036, China; State Key Laboratory of Tea Plant Biolog and Utilization, Anhui Agricultural University, 130 West Changjiang Road, Hefei 230036, China; State Key Laboratory of Tea Plant Biolog and Utilization, Anhui Agricultural University, 130 West Changjiang Road, Hefei 230036, China; State Key Laboratory of Tea Plant Biolog and Utilization, Anhui Agricultural University, 130 West Changjiang Road, Hefei 230036, China; State Key Laboratory of Tea Plant Biolog and Utilization, Anhui Agricultural University, 130 West Changjiang Road, Hefei 230036, China; State Key Laboratory of Tea Plant Biolog and Utilization, Anhui Agricultural University, 130 West Changjiang Road, Hefei 230036, China; State Key Laboratory of Tea Plant Biolog and Utilization, Anhui Agricultural University, 130 West Changjiang Road, Hefei 230036, China; State Key Laboratory of Tea Plant Biolog and Utilization, Anhui Agricultural University, 130 West Changjiang Road, Hefei 230036, China; Biotechnology of Natural Products, Technische Universität München, Liesel-Beckmann-Str. 1, 85354 Freising, Germany; State Key Laboratory of Tea Plant Biolog and Utilization, Anhui Agricultural University, 130 West Changjiang Road, Hefei 230036, China

## Abstract

Geraniol is an important contributor to the pleasant floral scent of tea products and one of the most abundant aroma compounds in tea plants; however, its biosynthesis and physiological function in response to stress in tea plants remain unclear. The proteins encoded by the full-length terpene synthase (*CsTPS1*) and its alternative splicing isoform (*CsTPS1*-*AS*) could catalyze the formation of geraniol when GPP was used as a substrate *in vitro*, whereas the expression of *CsTPS1*-*AS* was only significantly induced by *Colletotrichum gloeosporioides* and *Neopestalotiopsis* sp. infection. Silencing of *CsTPS1* and *CsTPS1*-*AS* resulted in a significant decrease of geraniol content in tea plants. The geraniol content and disease resistance of tea plants were compared when *CsTPS1* and *CsTPS1*-*AS* were silenced. Down-regulation of the expression of *CsTPS1*-*AS* reduced the accumulation of geraniol, and the silenced tea plants exhibited greater susceptibility to pathogen infection than control plants. However, there was no significant difference observed in the geraniol content and pathogen resistance between *CsTPS1*-silenced plants and control plants in the tea plants infected with two pathogens. Further analysis showed that silencing of *CsTPS1*-*AS* led to a decrease in the expression of the defense-related genes *PR1* and *PR2* and SA pathway-related genes in tea plants, which increased the susceptibility of tea plants to pathogens infections*.* Both *in vitro* and *in vivo* results indicated that *CsTPS1* is involved in the regulation of geraniol formation and plant defense via alternative splicing in tea plants. The results of this study provide new insights into geraniol biosynthesis and highlight the role of monoterpene synthases in modulating plant disease resistance via alternative splicing.

## Introduction

Tea (*Camellia sinensis*) is an important woody economic crop [1], and its leaves can be used to produce one of the world’s most important beverages [2]. Tea plants are susceptible to attack by various pathogens and insects during their growth [3]. Tea anthracnose disease caused by fungi in the genus *Colletotrichum*, especially *Colletotrichum gloeosporioides* [4] and gray blight disease caused by *Pestalotiopsis* species [5], are two of the most destructive foliar diseases of tea plants and are responsible for 30–60% [6] and 10–20% of the losses of tea products on an annual basis, respectively [5, 7]. Plants have evolved complex defense mechanisms to defend against pathogens [8]. Plant hormones such as salicylic acid (SA) and jasmonic acid play key roles in defense against pathogens [9]. SA is the primary hormone responsible for plant disease resistance, including the activation of the defense response following pathogen infection [6]. Previous studies have shown that the release of volatile terpenes is one of the key mechanisms by which plants resist pathogen [9].

Tea possesses abundant secondary metabolites that are strongly associated with its quality and health benefits [1, 10]. The release of defense-related volatiles plays an important role in mediating both local and systemic responses, as the emission of volatiles primes their defense mechanisms in response to attack by herbivores and pathogens [10–14]. The exposure of susceptible cultivars to volatiles from resistant cultivars can significantly increase the expression of defense-related genes and confer disease resistance [9, 15, 16]. Terpenoids contribute to tea flavor via their low human odor perception thresholds [17]. Monoterpenes, including linalool and geraniol, enhance the flavor and aroma of tea [18]. Linalool and geraniol are two of the most abundant and odor-active monoterpenoids in tea plants, and they contribute to the pleasant floral scent of tea products [17, 19]. Although the biosynthesis of the terpenoid pathway in tea plants has been studied, only a few terpene synthases (TPSs) and *TPS* genes involved in terpenoid synthesis have been identified [20]. The key gene involved in linalool formation in tea plants has been isolated and functionally verified [21]. However, the key enzyme involved in geraniol biosynthesis and its biological function in tea plants remains unclear [22].

Alternative splicing (AS) can generate different mRNA splicing isoforms from a single mRNA precursor via different splicing sites [23], and this can result in diverse protein isoforms [24]. An increasing number of studies have shown that AS plays an important role in the growth, development, and abiotic and biotic stress tolerance of plants [25, 26]. AS is also key in the biosynthesis of secondary metabolites [27] and the response to pathogen infection [28]. AS also figures prominently in abiotic stress tolerance, especially in ABA-mediated responses [24]. More than 41% of genes undergo AS during cold acclimation, and the four main types of AS events in tea plants are intron retention, exon skipping, alternative 5′ splice site, and alternative 3′ splice site [23] . AS isoforms of the *CsLOX2, CsLOX9*, and *CsLOX10* genes can be induced under low-temperature treatment [29]. AS in tea plants plays an important role in regulating the synthesis of secondary metabolites [30], including the synthesis of anthocyanins [31], linalool [21], and volatile fatty acid derivatives [32]. However, whether AS plays a role in the regulation of geraniol formation and biotic stress responses in tea following pathogen infection remains unclear.

Here, the first geraniol synthase (*CsGES*) was identified, cloned, and functionally characterized in tea plants. The expression level of the AS isoform *CsTPS1*-*AS*, but not the full-length *CsTPS1*, was significantly increased following *C. gloeosporioides* and *Neopestalotiopsis* sp. infection, and the function of *CsTPS1*-*AS* in planta was assessed. Silencing of *CsTPS1*-*AS* led to a decrease in the expression of defense-related and SA biosynthesis-related genes and an increase in the susceptibility of tea plants to *C. gloeosporioides* and *Neopestalotiopsis* sp. infection. The findings of this study enhance our understanding of geraniol formation in tea plants following fungal infection and provide new insights into the functions of AS isoforms during pathogen infection in plants.

## Results

### Geraniol synthase candidates identified by analysis of gene expression levels and geraniol accumulation in tea plants

**Figure 1:**
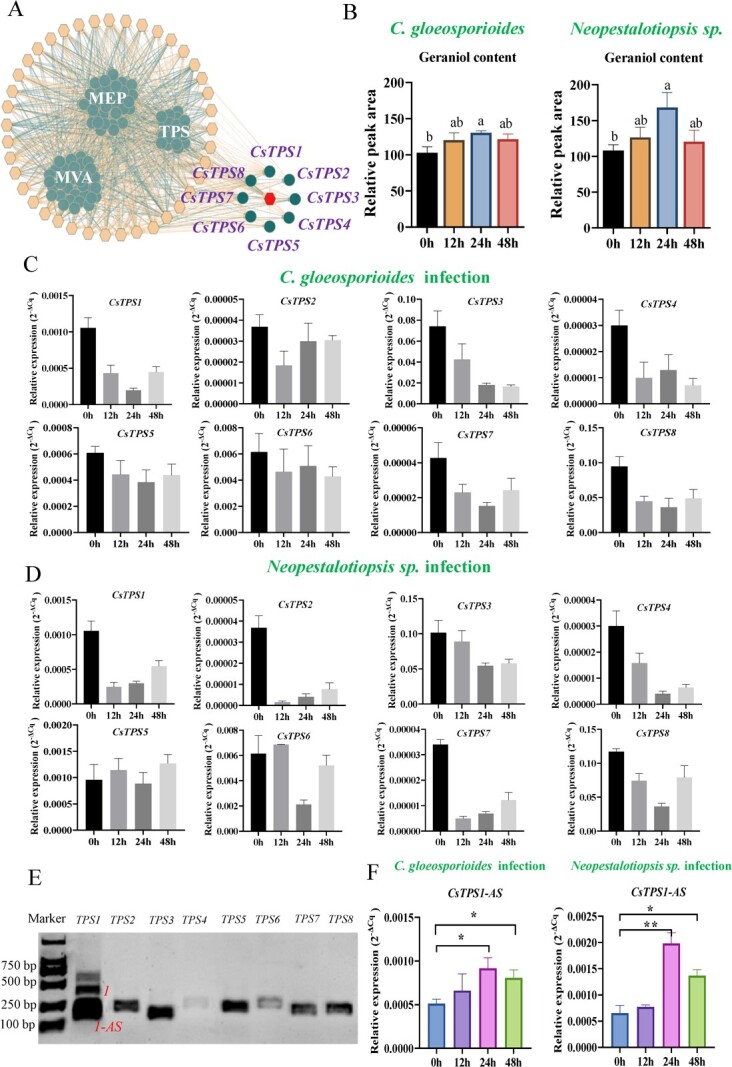
(**A**) Screening of a possible geraniol synthase gene (*CsGES*) of tea plants. The 41 yellow hexagons outside the large circle represent the 41 volatile terpenoids in tea plants; the one red hexagon represents geraniol; and the eight dark green dots around the red hexagon represent the eight *CsTPS* genes positively associated with geraniol formation. (**B**) Change in the geraniol content in tea plants infected with two fungal pathogens. (**C**, **D**) Expression of *CsTPS1–8* in response to pathogen-infected tea plants at different times following infection with *Colletotrichum gloeosporioides* and *Neopestalotiopsis* sp. (**E**) Verification of the specificity of the primers of eight candidate genes (*CsTPS1–8*) in infected tea plants with *Neopestalotiopsis* sp. after 24 h and qPCR products by agarose gel electrophoresis. (**F**) Expression of *CsTPS1*-*AS* (alternative splicing form of *CsTPS1*) in response to pathogen infection in tea plants at different times following infection with *C. gloeosporioides* and *Neopestalotiopsis* sp. Note: Letter codes indicate significant differences in geraniol content at *P* < 0.05 level indicated by Tukey’s analysis. ^*^ and ^**^ above columns indicate significant differences compared to Control under 5% and 1% levels of significance, respectively.

We identified *TPS* genes in tea plants from recently published tea genome sequences in the Tea Plant Information Archive (TPIA, http://tpia.teaplants.cn). Gene expression levels and terpenoid abundances permitted the identification of geraniol synthase (*CsGES*) genes in tea plants. According to the data we reported previously [33], there are 41 terpenoids and 27 TPS related genes differentially accumulated in five tissues (first leaf, second leaf, third leaf, mature leaf and stem), moreover, the significant correlation networks were generated by integrate the RPKM (reads per kilobase per million) value of 27 TPS related genes (dark green circle) and the content of 41 terpenoids (orange hexagon) using Pearson’s correlation analysis (r > 0.8 or r < −0.8, *P* < 0.05; left panel of Fig. 1A). To identify the geraniol synthase, we focused on the eight TPS genes that positively correlated with the geraniol content in the five tissues (first leaf, second leaf, third leaf, mature leaf, and stem) of tea plants, and listed them as geraniol synthase candidates (CsTPS1–CsTPS8) (indicated by dark green dots in the right panel of Fig. 1A) then the eight geraniol synthase candidates (*CsTPS1–CsTPS8*) positively associated with the geraniol content were selected for further study.

### Expression levels of eight candidate *CsGES* genes in pathogen-infected tea plants

Given that geraniol has been reported to function as an antifungal compound [34–36], changes in the abundance of geraniol in response to *C. gloeosporioides* and *Neopestalotiopsis* sp. infection were characterized using GC–MS. The geraniol content in the infected leaves significantly increased after 24 and 48 h of infection (Fig. 1B), indicating that geraniol might play a role in activating defense-signaling pathways following fungal attack in tea plants. To determine which candidates are involved in the biosynthesis of geraniol, gene-specific primers (Table S1, see online supplementary material) of these eight genes were designed, and the expression of these genes in response to pathogen infection was analysed 0, 12, 24, and 48 h after infection with *C. gloeosporioides* and *Neopestalotiopsis* sp. (Fig. 1C and D). To verify the specificity of the primers, the abundances of the transcripts of the eight candidate genes were analysed, and their products were verified by agarose gel electrophoresis (Fig. 1E). One clear band was observed for seven genes (*CsTPS2–CsTPS8*), whereas three clear bands were observed for *CsTPS1* (Fig. 1E), which indicates the presence of an AS form of *CsTPS1* in tea plants that is expressed in response to fungal attack.

**Figure 2:**
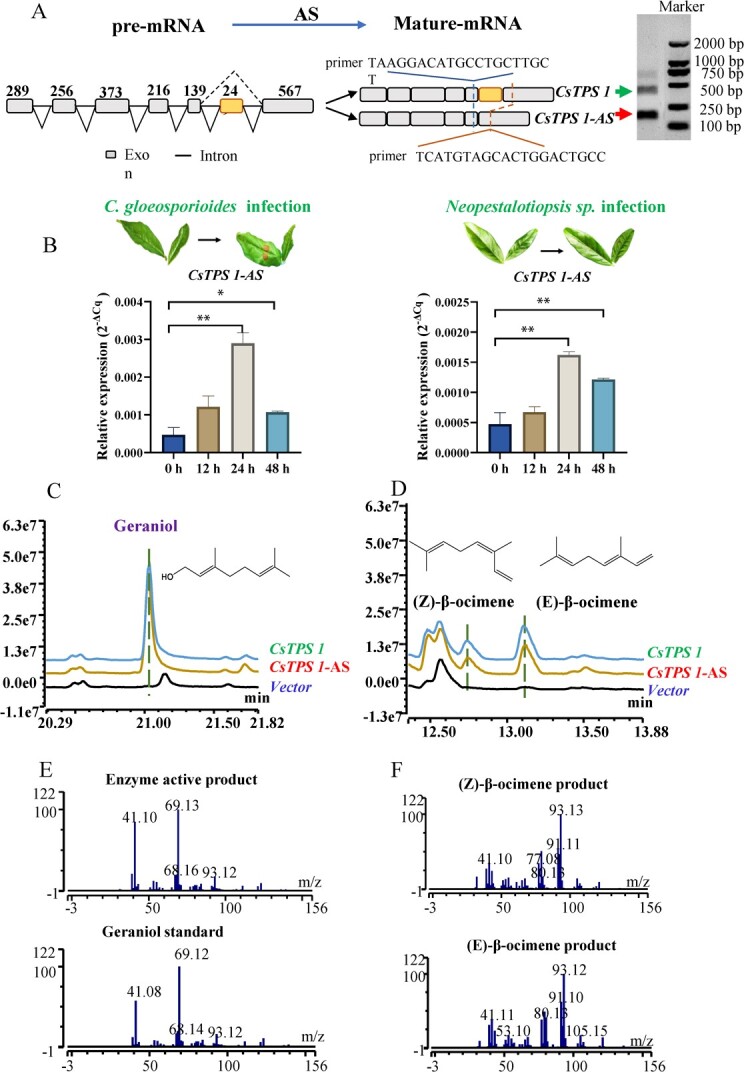
(**A**) AS isoforms of *CsTPS1* under pathogen infection in tea plants. (**B**) Expression of *CsTPS1-AS* in tea plants infected with two pathogens. (**C**, **D**) GC–MS analysis of the products formed by recombinant CsTPS1 and CsTPS1-AS enzyme *in vitro*. (**E**) The geraniol ion peak of CsTPS1 and CsTPS1-AS enzyme products and geraniol standard. (**F**) (Z)-β-ocimene and (E)-β-ocimene ion peaks of CsTPS1 and CsTPS1-AS enzyme products.

To verify the presence of the AS forms of *CsTPS1,* the full-length sequences and the shorter AS forms of *CsTPS1* were obtained from young leaves of *C. sinensis var. sinensis cv.* Shuchazao using gene-specific primer pairs (Table S1, see online supplementary material) [37, 38]. The whole-length *CsTPS1* contains a 1758-bp open reading frame (Fig. S1, see online supplementary material) that encoded 585 amino acids (Fig. S2, see online supplementary material); there were 83 fewer amino acids in the AS form (referred to as *CsTPS1-AS*) (Fig. 2A; Fig. S2, see online supplementary material). The AS form of *CsTPS1* was confirmed based on an AS database for tea plants (TeaAS, http://www.teaas.cn/index.php) [25]. The expression of *CsTPS1* and its AS isoform (*CsTPS1*-*AS*) was quantified in response to pathogen infection. To further verify whether *CsTPS1*-*AS* is expressed in tea plants in response to pathogen infection, the new specific quantitative primers for *CsTPS1*-*AS* and *CsTPS1* were redesigned (Table S1and Fig. S2, see online supplementary material). The expression of *CsTPS1*-*AS* and *CsTPS1* was quantified using RT-PCR, respectively. With the exception of *CsTPS1*-*AS*, the expression of none of the eight candidates was induced in tea plants following pathogen infection (Fig. 1C, D and F). The expression of *CsTPS1-AS* was significantly induced in response to infection with both *C. gloeosporioides* and *Neopestalotiopsis* sp.*,* which is consistent with changes in the content of geraniol in infected leaves. Therefore, the roles of *CsTPS1* and its AS forms in geraniol biosynthesis and the response to pathogen infection were studied.

To verify that *CsTPS1-AS* is involved in regulating geraniol biosynthesis and disease resistance in tea plants*,* the expression of *CsTPS1*-*AS* in infected tea plants was determined at various points after infection in repeated experiment (Fig. 2B). The expression of *CsTPS1-AS* was significantly increased under pathogen infection compared with the control, especially at 24 and 48 h, which is consistent with changes in the content of geraniol in leaves infected with the two pathogens (Fig. 1B). Overall, these findings indicate that *CsTPS1*-*AS* might be involved in the biosynthesis of geraniol in response to pathogen infection in tea plants.

### *CsTPS1* and its AS forms can catalyze the formation of geraniol *in vitro*

To determine whether *CsTPS1* and its AS form *CsTPS1*-*AS* are involved in the formation of geraniol in tea plants, *CsTPS1* and its AS splicing form *CsTPS1*-*AS* were expressed in *Escherichia coli* Rosetta (DE3) cells, and the enzymatic activity of the recombinant proteins was assessed using GPP as substrate. The products of the enzymes were adsorbed by SPME during the reaction process, and GC–MS was used to analyse the enzyme products. The recombinant proteins of *CsTPS1* and its AS splicing forms were involved in monoterpene formation when GPP was used as substrate (Fig. 2). The main product was identified as geraniol based on commercial standards; however, (E) β-ocimene and (Z) β-ocimene were also observed (Fig. 2C and D). No products were identified when FPP was used as the substrate. These *in vitro* data suggest that *CsTPS1* and its AS forms are involved in the formation of geraniol in tea plants.

### Geraniol inhibits the mycelial growth of fungi *in vitro*

**Figure 3:**
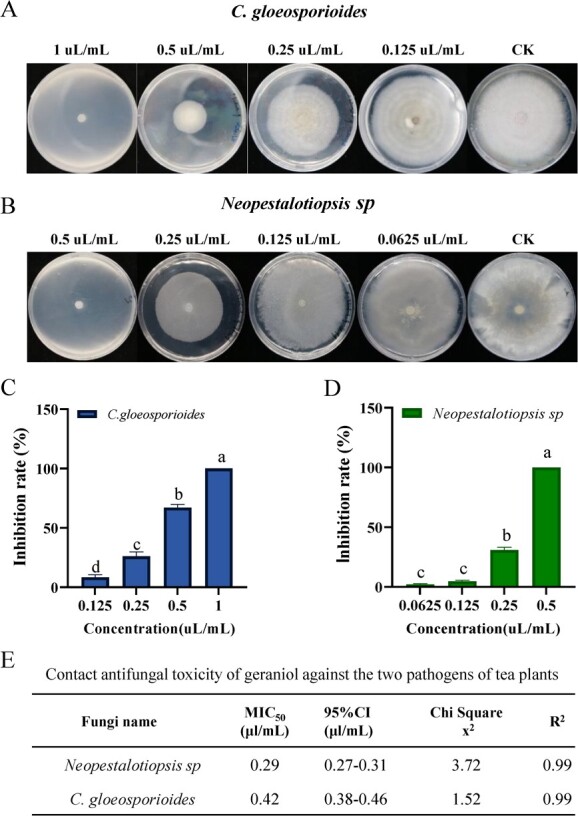
The antifungal activity of geraniol against the mycelial growth of *Neopestalotiopsis* sp. and *C. gloeosporioides in vitro*. (**A**, **B**) The mycelial growth of *C. gloeosporioides* and *Neopestalotiopsis* sp. under different concentrations of geraniol PDA medium. (**C**, **D**) The inhibition rate of different concentrations of geraniol against *C. gloeosporioides* and *Neopestalotiopsis* sp. (**E**) Contact antifungal toxicity of geraniol against *Neopestalotiopsis* sp. and *C. gloeosporioides*. Letters indicate significant differences among treatments (ANOVA, *P* < 0.05).

Experiments were carried out to evaluate the ability of geraniol to inhibit the growth of *Neopestalotiopsis* sp. and *C. gloeosporioides in vitro*. Geraniol inhibited the mycelial growth of the two pathogenic fungi. The mycelial growth of both fungi was dose-dependent *in vitro* (Fig. 3). Geraniol concentrations from 0.125 μL/mL to 1.0 μL/mL limited the mycelial growth of *C. gloeosporioides* (Fig. 3A and C). The mycelial growth of *Neopestalotiopsis* sp. was strongly inhibited by geraniol concentrations from 0.0625 μL/mL to 0.5 μL/mL (Fig. 3B and D). In addition, the MIC_50_ of geraniol against *Neopestalotiopsis* sp*.* and *C. gloeosporioides* was 0.29 μL/mL and 0.42 μL/mL, respectively (Fig. 3E), indicating that geraniol more strongly inhibited the mycelial growth of *Neopestalotiopsis* sp. compared with *C. gloeosporioides*.

### Silencing of *CsTPS1* and *CsTPS1-AS* reduces the geraniol content and pathogen resistance of tea plants

**Figure 4:**
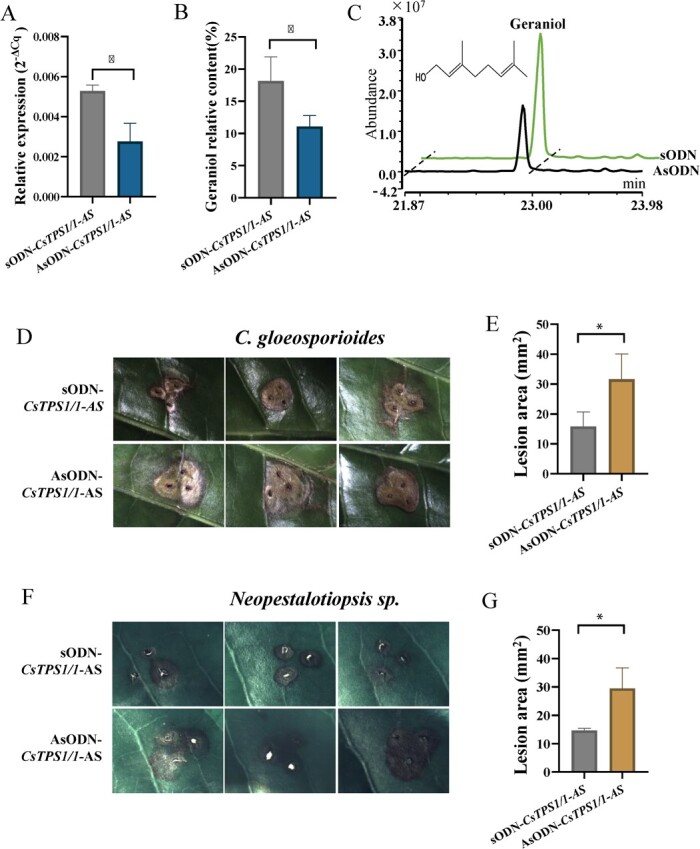
Functional analysis of *CsTPS1/1-AS* in tea plants. (**A**) The relative expression level of *CsTPS1/1-AS* in tea leaves treated with AsODN- *CsTPS1/1-AS* and sODN-*CsTPS1/1-AS* after 24 h. (**B**) The content of geraniol in tea leaves treated with AsODN-*CsTPS1/1-AS* and sODN-*CsTPS1/1-AS* after 24 h. (**C**) The total ion chromatograms of the geraniol content in tea leaves treated with AsODN-*CsTPS1/1-AS* and sODN-*CsTPS1/1-AS* after 24 h. (**D**, **E**) Disease symptoms of *C. gloeosporioides*-infected tea leaves of *CsTPS1/1-AS*-silenced and control tea plants after 72 h. (**F**, **G**) Disease symptoms of *Neopestalotiopsis* sp.-infected tea leaves of *CsTPS1/1-AS*-silenced and control tea plants after 72 h. Asterisks indicate significant differences among treatments (ANOVA,^*^P < 0.05).

The expression of *CsTPS1 and CsTPS1-AS* was simultaneously suppressed in tea leaves using a shared AsODN according to a previously described procedure [38]. The expression of *CsTPS1/1-AS* transcripts in tea leaves treated with AsODN-*CsTPS1/1-AS* for 24 h was significantly reduced compared with that in the control leaves (Fig. 4A). Consistent with the gene expression patterns, the abundance of geraniol was significantly reduced in *CsTPS-*silenced leaves compared with control leaves (Fig. 4B and C), indicating that *CsTPS1/1-AS* plays a key role in the formation of geraniol in tea plants.

Because the content of geraniol was increased in response to pathogen infection, we asked whether the formation of geraniol mediated by *CsTPS1/1-AS* plays a role in pathogen infection. To address this question, we silenced the expression of *CsTPS1/1-AS* in tea leaves. Subsequently, both the silenced and control tea leaves were infected with *C. gloeosporioides* and *Neopestalotiopsis* sp. The leaves of *CsTPS1/1-AS-*silenced and control tea plants showed typical disease symptoms 72 h post-infection (hpi) (Fig. 4D and F). However, the average surface area of disease spots in *CsTPS1/1-AS-*silenced leaves was significantly larger than that in control leaves (Fig. 4E and G). These results suggested that tea leaves became more susceptible to infection to both fungi when *CsTPS1/1-AS* was silenced. Overall, our results indicate that *CsTPS1/1-AS* plays a key role in the biosynthesis of geraniol and pathogen resistance of tea plants.

### *CsTPS1* and its AS forms confer different levels of disease resistance

**Figure 5:**
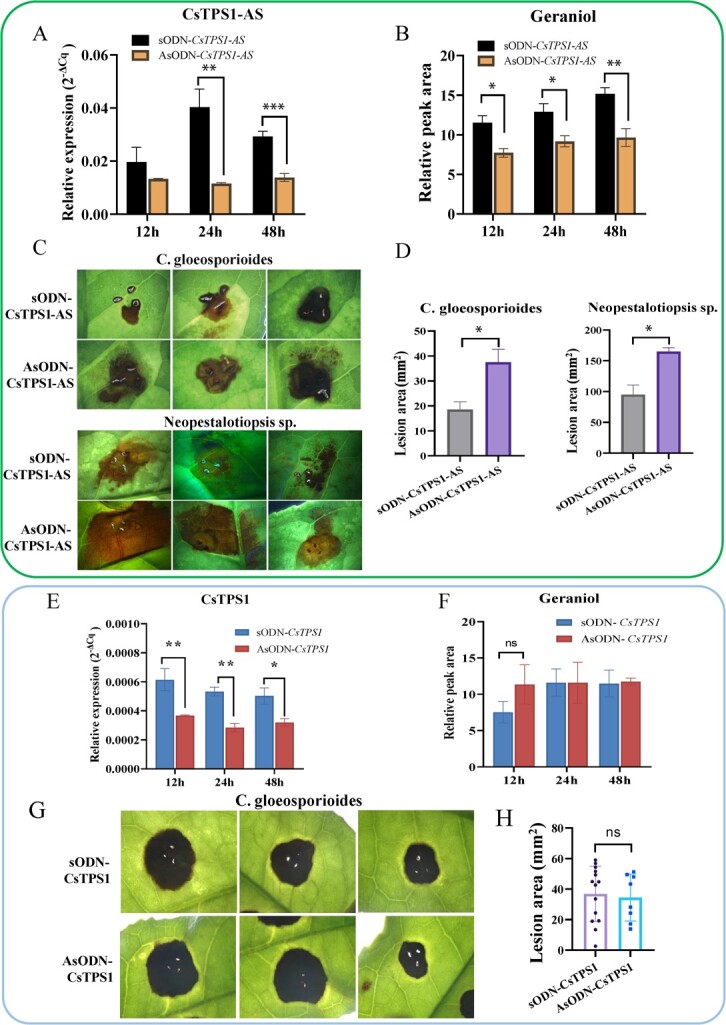
Functional analysis of *CsTPS1-AS* and *CsTPS1* in tea plants, respectively. (**A**) Relative expression level of *CsTPS1-AS* in tea leaves treated with AsODN-*CsTPS1-AS* and sODN-*CsTPS1-AS* at different times after infection. (**B**) The content of geraniol in tea leaves treated with AsODN-*CsTPS1-AS* and sODN-*CsTPS1-AS* at different times after infection. (**C**) Disease symptoms of *C. gloeosporioides* and *Neopestalotiopsis* sp.-infected tea leaves of *CsTPS1-AS*-silenced and control tea plants after 72 h. (**D**) Average surface area of disease spots in *CsTPS1-AS*-silenced leaves infected with *C. gloeosporioides* and *Neopestalotiopsis* sp. for 72 h. (**E**) Relative expression level of *CsTPS1* in tea leaves treated with AsODN-*CsTPS1* and sODN-*CsTPS1* at different times after infection. (**F**) The content of geraniol in tea leaves treated with AsODN-*CsTPS1* and sODN-*CsTPS1* at different times after infection. (**G**) Disease symptoms of *C. gloeosporioides*-infected tea leaves of *CsTPS1*-silenced and control tea plants 72 h after infection. (**H**) Average surface area of disease spots in *CsTPS1* -silenced leaves infected with *C. gloeosporioides* for 72 h. Asterisks indicate significant differences among treatments (ANOVA,^*^P < 0.05,^**^P < 0.01).

To compare the function of *CsTPS1* and its AS forms in regulating geraniol formation and pathogen resistance in tea plants**,** gene-specific AsODNs were designed to silence *CsTPS1* and its AS forms (Table S1, see online supplementary). The geraniol content was lower in tea leaves in which the expression of *CsTPS1-AS* was suppressed compared with that in control plants at 12, 24, and 48 h, respectively (Fig. 5A and B). As expected, *CsTPS1*-*AS*-silenced tea plants were more susceptible to infection with both *C. gloeosporioides* and *Neopestalotiopsis* sp. (Fig. 5C) at 72 hpi, as the average surface area of disease spots on the tea leaves was larger in *CsTPS1-AS-*silenced tea plants compared with that in control plants (Fig. 5D). By contrast, when *CsTPS1* was successfully suppressed in tea leaves (Fig. 5E), the geraniol content was not changed in tea leaves (Fig. 5F) in which the expression of *CsTPS1* was suppressed compared with that in control plants at 12, 24, and 48 h, respectively. Meanwhile, no difference in the susceptibility of tea leaves to pathogen infection was observed between *CsTPS1*-silenced tea leaves and control tea leaves at 72 hpi (Fig. 5G and H).

**Figure 6:**
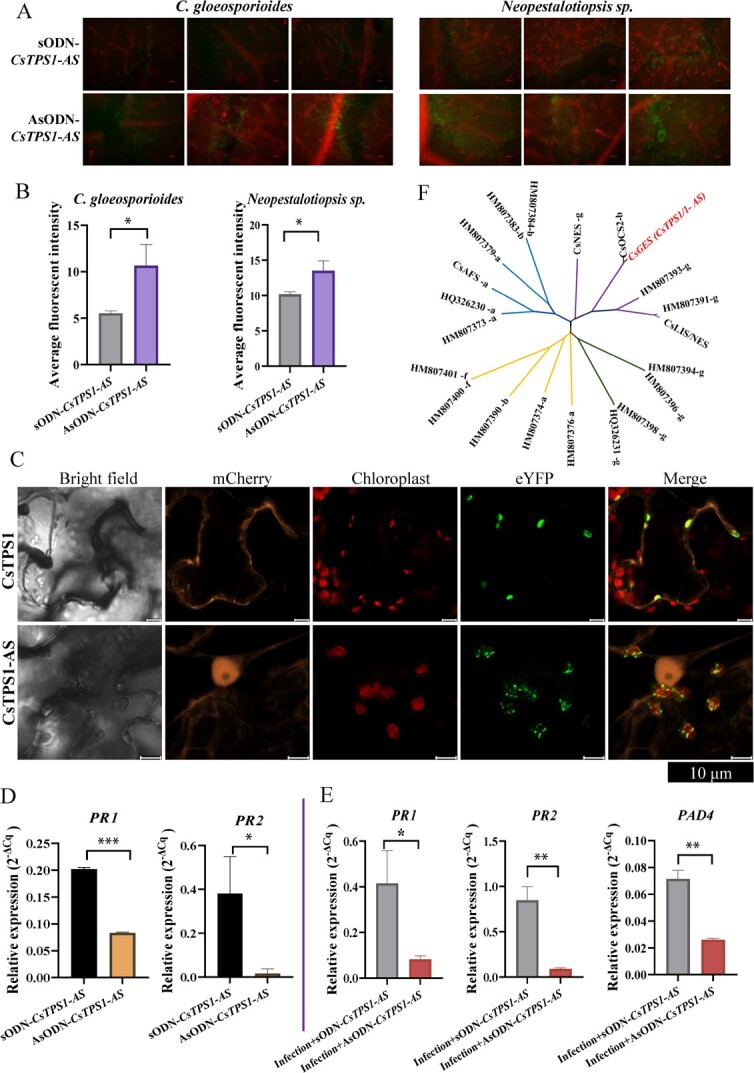
(**A**) WGA staining of the hyphal growth of *C. gloeosporioides* and *Neopestalotiopsis* sp. in *CsTPS1-AS*-silenced and control tea plants. (**B**) The green fluorescence intensity of *CsTPS1-AS*-silenced and control tea plants. (**C**) Subcellular localization of CsTPS1 and CsTPS1-AS proteins. (**D**) Expression level of *PR1* and *PR2* in *CsTPS1-AS*-silenced leaves and control tea leaves. (**E**) Expression of *PR1*, *PR2*, and *PAD4* in *C. gloeosporioides* infection tea leaves with AsODN-*CsTPS1-AS* and sODN-*CsTPS1-AS* treatment. (**F**) Phylogenetic tree of CsGES (CsTPS1\1-AS), CsAFS, α-farnesene synthase; CsOCS, β-ocimene synthase; CsNES, nerolidol synthase; and CsLIS/NES, linalool/nerolidol synthase: the other genes are from *Vitis vinifera*. Asterisks indicate significant differences among treatments (ANOVA,^*^P < 0.05,^**^P < 0.01,^***^P < 0.001).

WGA staining was used to observe the hyphal growth of *Neopestalotiopsis* sp. and *C. gloeosporioides* on tea leaves. After WGA staining, the hyphae emitted a green fluorescence under the microscope. The green fluorescence intensity of *CsTPS1-AS*-silenced tea leaves was higher than that of control tea leaves (Fig. 6A). The extent of mycelial growth on *CsTPS1-AS*-silenced tea leaves was higher than that on control leaves (Fig. 6B).

These findings indicate that *CsTPS1* and its AS forms perform distinct functions in both geraniol formation and pathogen resistance in tea plants and that *CsTPS1* plays a role in regulating geraniol biosynthesis and pathogen resistance via AS.

### The distribution and subcellular localization of CsTPS1 and CsTPS1-AS differ

Monoterpenes are synthesized exclusively by plastids in higher plants; thus, plant monoterpene synthases are localized to the chloroplast. To verify this prediction, the two CsTPS1 and CsTPS1-AS proteins were fused to the N-terminal of eYFP, and the fusion proteins were transiently expressed in tobacco leaves. The eYFP signals of CsTPS1 and CsTPS1-AS fusion proteins were consistent with chlorophyll autofluorescence and showed no overlap with the cytosolic mCherry signals from the negative controls (Fig. 6C). These findings confirmed that the two CsTPS1 and CsTPS1-AS proteins are localized to the chloroplast. However, the distribution and localization of the CsTPS1 and CsTPS1-AS proteins in the chloroplast varied (Fig. 6C). The CsTPS1 protein is likely localized in the stroma of the chloroplast and exhibits a highly homogeneous distribution. Conversely, the CsTPS-AS protein might be localized to the outer membrane of the chloroplast and exhibit a sporadic distribution (Fig. 6C). The distribution and localization of *CsTPS1* and its AS forms in the chloroplast differ, and this might explain the distinct levels of disease resistance that they confer to tea plants.

### *CsTPS1*-AS affects the expression of defense-related genes in the SA pathway in infected tea leaves

In plants, SA plays a crucial signaling role in activating defense pathways in plants, including systemic acquired resistance (SAR) and related immune responses. To verify the role of *CsTPS1-AS*-mediated disease resistance via activation of the expression of downstream-related defense genes in the SA pathway, we characterized the expression of defense-related genes in the SA pathway in *CsTPS1*-*AS*-silenced tea plants and control tea plants. The expression of *PR1* and *PR2* in CsTPS1-*AS-*silenced tea leaves was significantly lower than that in control plants (Fig. 6D).

*PAD4* (*Phytoalexin-deficient 4*) is an important signaling gene involved in activating the expression of downstream-related defense genes in the plant immune system. The expression of *PAD4* was not detected in control tea plants; however, its expression was significantly increased in infected tea plants (Fig. 6E). The expression of *PR1* and *PR2* in tea plants infected with *C*. *gloeosporioides* was approximately 2-fold higher than that in uninfected control tea plants (Fig. 6D and E). When tea leaves were infected with *C. gloeosporioides*, the expression of *PR1*, *PR2*, and *PAD4* was significantly reduced in *CsTPS1-AS*-silenced tea leaves compared with control plants (Fig. 6E). Overall, these findings indicate that *CsTPS1-AS* can affect the expression of genes in the SA pathway in infected tea leaves.

## Discussion

### CsTPS1 is a geraniol synthase in tea

Geraniol has a sweet, floral aroma similar to that of roses, and it contributes to the characteristic floral aroma and flavor of many fruits. Tea plants are important evergreen crops that are grown in temperate and subtropical regions. In response to herbivore and pathogen invasion, tea plants release volatiles, such as 3-hexenol, geraniol, β-ocimene, β-caryophyllene, and α-farnesene [39]. Tea green leafhopper, a major pest of tea plants, can significantly induce the emission of geraniol from tea leaves [39]. Other studies have shown that the higher content of geraniol in tea plants might be responsible for their stronger resistance to the pathogen causing tea leaf blight [40]. In addition, geraniol is considered one of the most abundant terpenes in tea, and it contributes greatly to its aroma [17]. Geraniol is an important defense-inducing substance in tea plants; however, the biosynthesis of geraniol in tea leaves has not yet been clarified.

Although geraniol synthase genes have been reported in *Vitis vinifera*, *Glycine max*, *Coffea arabica*, and other plants [41, 42], geraniol synthase genes have not yet been identified in tea plants. Only a few *TPS* genes have been identified in tea trees to date [22], such as *CsNES*, nerolidol synthase [20], *CsLIS/NES*, linalool/nerolidol synthase [21], *CsAFS* and α-farnesene synthase [43], *CsOCS* and β-ocimene synthase [44]. *CsTPS1* was first identified by analysis of gene expression levels and geraniol accumulation in tea plants, and both *in vitro* and *in vivo* analysis showed that it functions as a geraniol synthase in tea plants (Figs 2C and4B).

Plant *TPS*s are divided into seven families (TPS-a to TPS-g) [45], although phylogenetic analyses of terpenes can provide insights into the function of TPSs. However, *TPS*s on the same branch might have different functions [39] . In our study, *CsGES* (*CsTPS1/1-AS*) and *CsOCS* were in the same branch (Fig. 6F); their homologous sequence alignments were similar, but their functions were quite different. Phylogenetic analysis showed that *CsTPS1* clustered with *CsOCS2*, which belongs to the *TPS-b* gene family (Fig. 6F). The *TPS-b* subfamily is the second largest in *C. sinensis*, and it includes approximately 37.5% of all *TPS* genes in tea [22]. *CsOCS* specifically catalyzes the synthesis of β-ocimene from GPP [44], and *CsGES* (*CsTPS1/1-AS*) catalyzes the conversion of GPP to both geraniol and β-ocimene, and mainly catalyzed the synthesis geraniol. Therefore, the latter gene encodes the main enzyme that catalyzes the synthesis of geraniol (Fig. 2C and D).

### *CsTPS1* is involved in regulating the defense response via AS in tea plants

The transcriptional regulation of *TPS* genes is critically important for volatile terpenoid biosynthesis [46]. The substrate and product specificity of *TPS*s can regulate terpenoid biosynthesis at the enzyme level [47]. In addition to regulating transcriptional processes such as splicing, *TPS* genes also regulate other complex aspects of transcription. AS, which produces multiple mRNA subtypes from a single gene, is widespread in plants and often produces a variety of transcripts with diverse functions [48]. The full-length sequences and short AS forms of *CsTPS1* were obtained from the young leaves of tea plants. Although both *CsTPS1* and its AS forms could catalyze the formation of geraniol *in vitro*, *CsTPS1* and its AS forms confer different levels of disease resistance. The expression of *CsTPS1-AS*, but not the full-length sequences of *CsTPS1*, was induced in response to pathogen infection (Fig. 5C and G). This might explain differences in the distribution and localization of CsTPS1 and CsTPS1-AS in the chloroplasts.

The silencing of *CsTPS1-AS* significantly decreased the content of geraniol and the resistance of tea plants to infection by the two pathogens (Fig. 5B and C); however, no changes in disease symptoms were observed when *CsTPS1* was silenced (Fig. 5G and H). Hence, the shorter AS form *of CsTPS1* plays a critical role in enhancing the resistance of tea plants to pathogen infection.

The alternative splicing plays an important role in plants’ response to biotic stress. AS of pre-mRNA is a crucial post-transcriptional regulatory mechanism to the generation of structurally variable transcripts from a single gene. AS events can increase transcriptome and proteome diversity and regulate transcript levels following transcription. A large number of studies have shown that AS has a range of physiological functions and plays an important role in plant development, growth, and biotic stress response [49]. In this study, although the enzyme activity of *CsTPS1* and its AS form is similar, only the AS isoform could be regulate by infection. It is possible mechanisms that AS of *CsTPS1* are probably pathogen-sensitive, whereas *CsTPS1* is essential for tea plant growth and development.

Current research on AS mostly focuses on plants’ biotic stress-response genes undergoing AS in infected plants by pathogens, thereby regulating plant immunity. Many biotic stress-response genes undergo alternative splicing in pants with pathogen infection. These biotic stress-response AS genes include disease resistance (R) genes [50, 51], receptor-like kinase [52], pathogen-induced transcription factor [53] and plant immunity-related genes [54]. The plant’s immune response is regulated by the AS of protein kinase genes. The *Calcium-Dependent Protein Kinase* 28 (*CPK28*) is a negative immune response regulator that targets BIK1 (Botrytis-Induced Kinase 1) for degradation [49]. However, the *CPK28-*AS isoform acts as a positive regulator of PTI (molecular patterns associated with pathogens (PAMP)-triggered immunity) [54]. Furthermore, there is still no clear understanding of how AS is triggered by pathogenic infections to inducedplant immunity [49].

However, an important question regarding whether and how plant pathogens target splicing in their host remains mostly unknown. Few studies have explained the potential mechanism by which plant pathogens regulate the occurrence of AS in infected plants. Very few studies have shown that pathogenic effectors of pathogens bind host plant pre-mRNA to manipulate the occurrence of AS of host pre-mRNA, thereby regulating host plant immunity. The wheat pathogenic fungus *Puccinia striiformis* (*Pst*) produces pathogenic ‘splicing’ effectors *Pst_A23*, which regulate host pre-mRNA splicing by directly binding the host plant pre-mRNA splice site, thereby interfering with host immunity [55]. Another study showed that the pathogen effectors of *Phytophthora infestans*, pathogenic fungus of tomato leaves, binds host mRNAs to manipulate the plant AS, leading to the reprogramming of plant immunity [56]. Combined with the above analysis, we speculate that it may also be pathogenic effectors binding the tea plant’s pre-mRNA of *CsTPS1* causing the occurrence of AS of *CsTPS1* in infected tea plants, although the exact mechanism needs to be further studied.

### *CsTPS1-AS* enhances the resistance of tea plants to pathogen infection by regulating geraniol formation and the expression of SA-related genes

Plant pathogens can activate SA pathways, which enhance the resistance of plants to pathogen infection [15]. The pathogenesis-related defense genes *PR1* and *PR2* are typical markers of the SA-mediated defense system [57]. The expression of *PR1* and *PR2* was significantly increased in pathogen-infected tea plants (Fig. 6D and E). This indicates that pathogens can induce the expression of pathogenesis-related genes in the SA-mediated pathway, which enhances the resistance of plants to pathogen infection. The expression of *PR1* and *PR2* was significantly lower in *CsTPS1-AS-*silenced plants than in control plants (Fig. 6D). This suggests that *CsTPS1-AS* mediates the response to pathogen infection by up-regulating the expression of pathogenesis-related genes.

*PAD4* is known to play a key role in SAR through SA-dependent and SA-independent pathways [58, 59]. To further clarify the role of *CsTPS1-AS* in plant defense, the expression of *PAD4* was assessed after pathogen infection in tea plants. As expected, silencing of *CsTPS1-AS* significantly decreased the expression of *PAD4* in infected tea plants (Fig. 6E), suggesting that *CsTPS1*-AS might enhance SAR in tea plants by activating the expression of *PAD4*. Overall, these findings indicate that *CsTPS1-AS* might play a role in pathogen resistance by regulating the expression of *PR1*, *PR2*, and *PAD4*.

**Figure 7:**
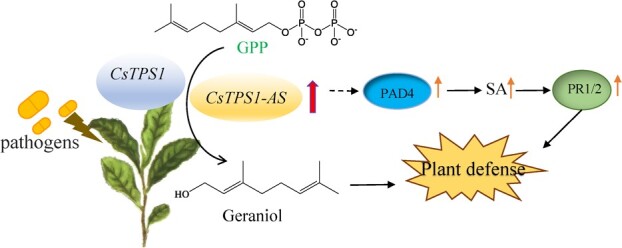
The working model for the function of *CsTPS1* to formulation the geraniol and enhances the resistance to pathogen infection via alternative splicing (*CsTPS1-AS*) in tea plants.

Silencing of *CsTPS1*-*AS* also significantly decreased the content of geraniol (Fig. 5B) and the amount of mycelial growth on CsTPS1-AS-silenced tea leaves was more than that on control leaves (Fig. 6A and B). Meanwhile, geraniol has shown to more strongly inhibit the mycelial growth of *Neopestalotiopsis* sp. and *C. gloeosporioides in vitro* (Fig. 3). These findings indicate that geraniol plays an important role in enhancing resistance to infection by both of these fungal pathogens. Our findings are consistent with the results of a previous study showing that (E)-β-caryophyllene mediates the defense response of *Arabidopsis thaliana* flowers to pathogen infection by directly inhibiting bacterial growth [60]. Our findings indicate that the function of *CsTPS1*-*AS* was to enhance the resistance of tea plants to pathogen infection by up-regulating the biosynthesis of geraniol. Thus, *CsTPS1*-*AS* enhances the resistance to pathogen infection in tea plants by regulating geraniol formation and the expression of SA-related genes. Based on these results, we propose a putative working model for the function of *CsTPS1/1*-*AS* in pathogen infection (Fig. 7).

## Conclusion

In conclusion, we identified a key *TPS* gene that functions as a geraniol synthase (*CsGES*) in tea plants, and both *in vitro* and *in vivo* studies indicated that this geraniol synthase is involved in regulating geraniol formation and plant defense via AS. The results of this study provide new insights into geraniol biosynthesis and clarify the role of monoterpene synthases in modulating the disease resistance in plants via AS.

## Materials and methods

### Plant material

Tea plants were gathered from the Tea Plant Cultivar and Germplasm Resource Garden at Anhui Agricultural University (Guohe Town, China) and promptly cryogenically preserved in liquid nitrogen. The entirety of the tea specimens were maintained at a temperature of −80°C until they were ready for utilization.

### Chemicals and reagents

Standards of geranyl pyrophosphate (GPP), farnesyl pyrophosphate (FPP), geraniol, (Z)-β-ocimene, and (E)-β-ocimene were purchased from Sigma-Aldrich (St Louis, MO, USA).

### Integration of TPS genes and terpenoid

RPKM gene values and the proportional terpenoid content were employed as a matrix for conducting Pearson’s correlation analysis, considering correlations where r > 0.8 or r < −0.8 and *P* < 0.05. The resultant correlation networks were derived and visually represented using Cytoscape software (version 2.6.3).

### RNA extraction and cDNA cloning

Total RNA was extracted from *C. sinensis* (SCZ) leaves utilizing the Fast Pure Plant Total RNA Isolation Kit (Vazyme, China) following the guidelines of the manufacturer. The cDNA was then synthesized through reverse transcription of the total RNA using the PrimeScript RT Master Mix (Vazyme, China). Primers for the cloned *CsTPS1/1*-*AS* gene are shown in Table S1 (see online supplementary material). The PCR products were purified using a Gel Extraction Kit (Vazyme, China). The resultant target cDNA fragment was inserted into the pGEX-4 T1 vector, followed by transformation into Trans 1-T1 competent cells.

### GC–MS analysis of geraniol and other volatiles in tea samples

Geraniol and other volatile compounds in the samples were examined using a combination of SPME and GC/MS (Thermo Scientific TRACE 1300, ISQ 7000, MA, USA). In brief, the tea samples were ground into powder in liquid nitrogen, 0.2 g of each sample was weighed and placed into the sample vial for testing. An internal standard of two μl of ethyl caprate (1 ppm in methyl alcohol) was introduced. The samples were then incubated at 60°C for 1 hour, during which the volatiles were absorbed by the SPME process. GC column: DB-5, 60 m × 0.25 mm, film thickness 0.25 μm (J&W Scientific, USA). Pure helium was used as the carrier gas at a flow rate of 1 mL/min. The GC injector had a split ratio of 10:1. The GC oven condition: maintained at 40°C for 3 min, increased by 5°C/min to 80°C; increased to 160°C at 2°C/min; and then to 240°C at a rate of 10°C/min; held at 240°C for 5 min. Full-scan mode with an m/z range of 300–600 was applied. All compounds were identified by comparison with a mass spectrometry library (NIST) and compounds with known retention times. Geraniol, (Z)-β-ocimene, and (E)-β-ocimene (Sigma-Aldrich Chemie GmbH, Taufkirchen, Germany) were identified using standards.

### Heterologous protein expression and purification

Heterologous protein expression and purification were carried out following the methods of a previous study [61] with slight modifications. The complete coding sequence of CsGES was enzymatically digested using BamH1 and Smal1, yielding gene fragments that were subsequently introduced into pGEX-4 T-1. The recombined plasmids were then transformed into *E. coli* strain BL21 (DE3) pLysS cells. Following incubation at 37°C for approximately 24 h until the optical density (OD_600_) of the cultured cells reached 0.6–0.8, then isopropyl-ß-D-thio-galactopyranoside was added with a final concentration of 1 mM and incubated at 16°C for 22 h to induce protein expression. The expressed protein was then isolated and refolded as described in a previous study [62]. The fusion proteins were purified by GST-binding resin following the manufacturer’s protocol. A photometric method was used to determine the protein concentration [63] with BSA as a standard. The correct size of the proteins was confirmed by SDS–PAGE.

### Enzyme assay for geraniol synthase (*CsGES*)

Enzyme activity assays were carried out in 1-mL reaction buffer within a 20 mL tube, reaction buffer: pH 7.2, 0.1 M PBS, 10 mM MgCl_2_, 1 mM MnCl_2_, 100 mM KCI, and 1 mM DTT, 10% glycerol (v/v), containing crude recombinant protein (50–100 μg) and substrate FPP/GPP (5 μg) [41]. The reactions were incubated at 30°C for 1 h and then at 42°C for 15 min [20], and the products were collected by SPME. At least three bioreplicates have been performed. The reaction products were identified using GC–MS per the method described above. Enzyme activity products, geraniol, (Z)-β-ocimene, and (E)-β-ocimene were identified using comparison standards.

### Gene suppression of *CsGES* in *C. sinensis* using AsODNs

Functional assays of *CsGES* (*CsTPS1*, *CsTPS1-AS*, and *CsTPS1/1*-*AS*) in tea plants were carried out by suppressing the expression of *CsGES* in *C. sinensis* following a previously described method [38]. Candidate sequences (Table S1, see online supplementary material) of the antisense oligonucleotide (AsODN) of target gens (*CsTPS1*, *CsTPS1-AS*, and *CsTPS1/1*-*AS*) were selected using Soligo software (http://sfold.wadsworth.org/cgi-bin/index.pl), respectively. By analysing the cDNA sequence, the specifically AsODNs primers were designed, and they can specifically silence the target gene (Fig. S3, see online supplementary material). AsODNs were synthesized by TSINGKE Biological Technology Co., Ltd (Anhui, China). The target gene in the tea leaves was silenced using AsODN following a previously described method [38, 64]. Briefly, 1 mL of 40 μM AsODN- *CsTPS1/1*-*AS* solution (to suppress both *CsTPS1* and *CsTPS1*-*AS*) and AsODN-*CsTPS1* solution (to suppress *CsTPS1*), or AsODN-*CsTPS1*-*AS* solution (to suppress *CsTPS1*-*AS*) was injected into whole tea leaves. The sense oligonucleotides (sODN) were injected into tea leaves as a control treatment. At least six experimental replicates were performed for each treatment. After treatment, the tea leaves were harvested, rapidly frozen using liquid nitrogen, then stored at −80°C before analysis. The content of geraniol was detected as described above.

### Quantitative real-time PCR analysis

For real-time PCR assay, total RNA from tea leaves was used as template, the specific primer sequences were listed in Table S1 (see online supplementary material). The glyceraldehyde-3-phosphate dehydrogenase (*GAPDH*) gene and *β-actin* were used as an internal reference gene, and relative expression levels were calculated using the 2^–ΔCT^ method according to our previous research [65, 66]. All reactions were carried out using the CFX96™ Real-Time System (Bio-Rad, USA). The temperature program: 95°C for 3 min, followed by 40 cycles of 95°C for 10 s and 62°C for 30 s.

### Pathogen cultivation and infection of tea plants

The pathogenic fungi *Neopestalotiopsis* sp. and *C. gloeosporioides* were cultivated in PDA medium in Petri dishes and grown in an incubator at 25°C ± 3 with a humidity of 75 ± 5%. The pathogenic infection experiment was carried out as follows. Briefly, one-year-old *C. sinensis* (SCZ) seedlings were selected, and the leaves in each treatment were wounded with a sterile needle. Five-mm diameter mycelial discs of *C. gloeosporioides* and *Neopestalotiopsis* sp. grown on PDA were inoculated into the test leaves. The leaves treated with 5-mm diameter pure PDA were set as control. Finally, the seedlings were grown in a greenhouse. At least six bioreplicates were performed.

### Contact antifungal activity of geraniol *in vitro*

The contact antifungal activity of geraniol against *Neopestalotiopsis* sp. and *C. gloeosporioides* was determined following a procedure described in a previous study [7]. Serial two-fold dilution method was applied to assess the MIC_50_ of geraniol. Drawing from initial trials, the initial solution underwent serial dilution in 30 mL of PDA medium at 45–50°C across various concentrations (1 μl/mL, 0.5 μl/mL, 0.25 μl/mL, 0.125 μl/mL, and 0.0625 μl/mL) to assess its inhibitory effect on *Neopestalotiopsis sp.* and *C. gloeosporioides*. The negative control was treated with an equivalent volume of acetone blended with PDA. Ten mL of toxic medium was poured into aseptic Petri dishes. A 5-mm diameter fungal disc of *Neopestalotiopsis* sp. and *C. gloeosporioides* was promptly inoculated at the center of each PDA plate. Subsequently, the plates were incubated in darkness at a temperature of 25°C. Following a 5-day incubation period, colony growth diameter was measured with digital calipers. Every test was replicated thrice.

### Pathogen infection of tea plants treated with AsODNs

*C. sinensis* (SCZ) leaves showing no signs of disease and insect damage were used in experiments. The gene suppression technique outlined earlier was employed to induce silencing of the target gene in each treated tea leaf. Briefly, AsODN- *CsTPS1/1*-*AS,* AsODN-*CsTPS1*, and AsODN-*CsTPS1*-*AS* solution was injected into the tea leaves of different treatments. The treated tea leaves were then immediately inoculated with mycelial discs (5 mm diameter) of *Neopestalotiopsis* sp. and *C. gloeosporioides*. In the control treatment, each treated tea leaf was injected with the equivalent volume of sODN solution and promptly inoculated with mycelial discs of the two pathogens. All treated tea plants were cultured in a greenhouse at 25 ± 3°C with 70 ± 5% relative humidity and a 16/8 hr (day/night) photoperiod. Treated tea leaves were collected for analysis after 72 h when they showed signs of disease. There were at least six biological replicates for each treatment.

### WGA staining and microscopic observation of pathogenic hyphae

The growth status of pathogens in tea leaves was assessed using a stereoscopic fluorescence microscope (Olympus SZX16, Tokyo, Japan), and the total infected area was measured using image analysis software (Olympus Cellsens Standard, Tokyo, Japan). Tea leaves inoculated with *Neopestalotiopsis* sp. and *C. gloeosporioides* were placed in 4-mL centrifuge tubes with FAA fixed solution (G1103, Servicebio®, Wuhan China); sent to Wuhan Seville Biotechnology Co., Ltd for fluorescent wheat germ agglutinin (WGA) staining; and photographed with a fluorescence microscope.

### Subcellular localization analysis of CsTPS1 and CsTPS1-AS proteins

Subcellular localization assays of CsTPS1 and CsTPS1-AS proteins were performed following the procedure described in a previous study [67]. Briefly, binary vectors (pCHNP-eYFP/mCherry) were constructed with several elements on the pCAMBIA1300 backbone (CAMBIA, Canberra, Australia). The amplified fragments were introduced into pCHNP-eYFP with the NcoI site using in-fusion technology. The empty vector pCHNP-mCherry was used as a negative control. *Agrobacterium tumefaciens* strain GV3101 carrying the construct for the transient expression of individual mCherry and CsTPS1 EYFP and CsTPS1-AS EYFP fusion proteins was mixed and infiltrated into the leaves of tobacco. Images were taken using a laser confocal fluorescent microscope (Lecia DMi8, Germany). The EYFP, mCherry fluorescence, and chloroplast autofluorescence were analysed at excitation wavelengths of 488 nm, 561 nm, and 561 nm and emission wavelengths of 500–530 nm, 580–620 nm, and 680–720 nm, respectively.

## Supplementary Material

Web_Material_uhad184

## Data Availability

All relevant data can be found within the paper and its supporting materials.
